# Electrocatalytic Assisted Performance Enhancement for the Na-S Battery in Nitrogen-Doped Carbon Nanospheres Loaded with Fe

**DOI:** 10.3390/molecules25071585

**Published:** 2020-03-30

**Authors:** Jianhui Zhu, Amr Abdelkader, Denisa Demko, Libo Deng, Peixin Zhang, Tingshu He, Yanyi Wang, Licong Huang

**Affiliations:** 1 College of Chemistry and Environmental Engineering, Shenzhen University, Shenzhen 518060, China; zhujhszu@163.com (J.Z.); wangyanyi1984@163.com (Y.W.); huanglc26@foxmail.com (L.H.); 2 College of Materials Science and Engineering, Xi’an University of Architecture and Technology, Xi’an 710055, China; hetingshu@xauat.edu.cn; 3 Department of Design and Engineering, Faculty of Science & Technology, Bournemouth University, Poole, Dorset BH12 5BB, UK; aabdelkader@bournemouth.ac.uk; 4 Department of Engineering, University of Cambridge, Cambridge CB3 0FS, UK; dd486@cam.ac.uk

**Keywords:** sodium-sulfur battery, catalytic activity, hollow N-doped carbon nanospheres, chemical absorption, polysulfides

## Abstract

Room temperature sodium-sulfur batteries have been considered to be potential candidates for future energy storage devices because of their low cost, abundance, and high performance. The sluggish sulfur reaction and the “shuttle effect” are among the main problems that hinder the commercial utilization of room temperature sodium-sulfur batteries. In this study, the performance of a hybrid that was based on nitrogen (N)-doped carbon nanospheres loaded with a meagre amount of Fe ions (0.14 at.%) was investigated in the sodium-sulfur battery. The Fe ions accelerated the conversion of polysulfides and provided a stronger interaction with soluble polysulfides. The Fe-carbon nanospheres hybrid delivered a reversible capacity of 359 mAh·g^−1^ at a current density of 0.1 A·g^−1^ and retained a capacity of 180 mAh·g^−1^ at 1 A·g^−1^, after 200 cycles. These results, combined with the excellent rate performance, suggest that Fe ions, even at low loading, are able to improve the electrocatalytic effect of carbon nanostructures significantly. In addition to Na-S batteries, the new hybrid is anticipated to be a strong candidate for other energy storage and conversion applications such as other metal-sulfur batteries and metal-air batteries.

## 1. Introduction

Future energy needs to be cost-effective with sustainable production, as well as amenable to proper storage for on-demand use [1,2,3,4,5,6]. A series of emerging applications such as electric vehicles and large-scale grids demand technologies to produce inexpensive and long cycle life energy storage systems (ESSs) [7,8,9,10,11,12,13,14]. Due to the high theoretical specific energy, environmental benignity and abundance of sulfur (S), the lithium-sulfur battery (Li-S battery) has attracted great attention [15,16,17]. Similar to the Li-ion battery, large-scale applications of Li-S batteries is restricted due to high cost and limited sources of lithium [18,19].

The room-temperature (RT) sodium-sulfur battery (Na-S battery), which shares a similar reaction mechanism to that of the Li-S battery, was initially reported in 2006 [20]. It is an ideal candidate to meet the future demands because of its notable merits including large energy density (theoretical value 1673 mAh·g^−1^), low material cost, and resource abundance [19,21,22,23]. During the charge and discharge process, a series of reactions occur as follows [24]:

(1) $$4\mathrm{Na}+S_{8}↔2{\mathrm{Na}}_{2}S_{4}\;E=2.03\;V$$

(2) $$2\mathrm{Na}+{\mathrm{Na}}_{2}S_{4}↔2{\mathrm{Na}}_{2}S_{2}\;E=2.03\;V$$

(3) $$2\mathrm{Na}+{\mathrm{Na}}_{2}S_{2}↔2{\mathrm{Na}}_{2}S\;E=1.68\;V$$

The multielectron electrochemical reactions between the sulfur (S) cathode and sodium (Na) anode contribute to the ultrahigh energy density. However, the RT Na-S battery still faces some critical challenges. First of all, the poor electric conductivity of S and the sluggish reactivity of S with Na result in capacity fading. More importantly, long-chain polysulfides are readily dissolved in liquid electrolytes and diffused across the liquid electrolyte to the anode. On the surface of the anode, these long-chain polysulfides undergo redox reaction and form short-chain sulfides, which also lead to severe capacity fading [25]. These challenges impede the development of a high-performance RT Na-S battery.

Considerable developments have been made in the RT Na-S battery since 2012 [21]. Extensive efforts, such as embedding the S species in a functional matrix and coating or modification of separator and passivation of the anode have been made to minimize the shuttle effect [26,27,28,29,30,31,32]. For example, Ma et al. designed a freestanding C/S/BaTiO_3_@TiO_2_ electrode and used it as the cathode for the RT Na-S battery [30]. The results indicated that the “BaTiO_3_-C-TiO_2_” synergetic structure ensured effective prevention of the shuttle effect, restraining the volumetric variation and stabilizing the ionic transport interface. Recently, a novel S host material combining carbon and electrocatalyst has been designed and investigated in the Li-S battery and the RT Na-S battery [33,34,35,36,37,38,39,40,41,42]. In particular, transition-metal based electrocatalysts, low-cost high-performance electrocatalysts [43,44], have already demonstrated a great potential in the development of the RT Na-S battery [33,35]. Such a strategy works on the basis that catalytic materials enhance the electrochemical redox kinetics by reducing electron and ion transfer resistance resulting in decreased solubility of the polysulfides in the organic electrolyte. Zhang et al. tested atomic cobalt (Co) (including single atom and nanoclusters) anchored on hollow carbon nanospheres and delivered remarkable cycling stability (507 mAh·g^−1^ after 600 cycles at 100 mA·g^−1^), as well as excellent rate performance (220.3 mAh·g^−1^ at 5 A·g^−1^) [33]. They also confirmed that Fe clusters support on hollow carbon nanospheres also displays high performance for the RT Na-S battery [35]. The result of theoretical calculation showed that the adsorption energy of Na_2_S and Na_2_S_4_ on Fe cluster was much stronger than those on a pure carbon support, which indicated that the decomposition of Na_2_S_4_ could be electrocatalyzed. It indicated that the host materials constructed by transition metal based electrocatalysts and functional carbonaceous materials provided a promising way to develop a high-performance RT Na-S battery.

Heteroatoms-doped carbons loaded with Fe ions, which have been extensively used as high performance and inexpensive catalysts for oxygen reduction reaction (ORR) [45,46], have also been studied as useful host material for the Li-S battery and RT Na-S battery [34,35]. Herein, we report an effective S host material with Fe ions loaded in a hollow N-doped carbon nanosphere (Fe@HNCS) obtained via a simple method. The hollow N-doped carbon nanosphere (HNCS) acts as a continuous conductive backbone, which offers a short diffusion path for ion and electron diffusion. In addition, Fe ions serve as catalytic sites for accelerating the conversion of long-chain polysulfides to short-chain polysulfides and possess a strong chemical interaction with soluble polysulfides. Thus, the Fe@HNCS electrode possesses better electrochemical performance as compared with the bare HNCS. Especially, the Fe@HNCS obtained at 800 °C delivers the highest reversible capacity of 359 mAh·g^−1^ at 0.1 A·g^−1^, a relatively stable capacity of 180 mAh·g^−1^ at 1 A·g^−1^ with almost 100% coulombic efficiency after 200 cycles and excellent rate performance.

## 2. Results and Discussion

**Figure 1:**
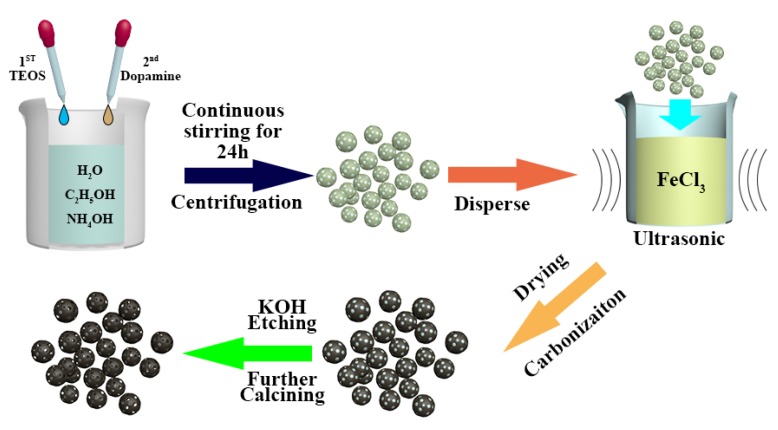
The synthesis route of Fe ions loaded in a hollow N-doped carbon nanosphere (Fe@HNCS) preparation.

The preparation procedure for Fe@HNCS is schematically shown in Figure 1. First, silica nanospheres were prepared and used as hard templates, which were, then, coated with polydopamine (PDA) by in situ polymerization of dopamine hydrochloride, forming a core-shell structure (PDA@SiO_2_). Then, the composite nanospheres were soaked in FeCl_3_ solution, through which the Fe^3+^ decorated uniformly on the surface of PDA@SiO_2_ due to the coordination between PDA and Fe ions. After impregnating with FeCl_3_, the composite was pyrolyzed at different temperatures. During this process, first, FeCl_3_ hydrolyzed to the amorphous Fe species in solutions including Fe(OH)_3_ and FeO(OH) below 350 °C. Then, amorphous Fe species converted into Fe_2_O_3_ below 400 °C [47]. On the one hand, at higher temperatures (500 to 700 °C), Fe_2_O_3_ reduced to Fe_3_O_4_. Then, Fe_3_O_4_ was converted to α-Fe through carbothermal reduction and formed liquid droplets below 800 °C. The carbon nanospheres, on the other hand, started the graphitization process at around 800 °C [48]. Next, the samples were leached by acid to remove large Fe particles which are catalytically inactive and harmful in that they densify the hybrid and block the diffusion of electrolyte in the catalyst [49]. Finally, a second calcination step was employed to improve the performance by removing the unstable molecules and expose more active sites [50]. The final product, i.e., hollow N-doped carbon nanospheres loaded with Fe ions, is denoted as Fe@HNCS-*T*, where *T* represents the pyrolysis temperature.

**Figure 2:**
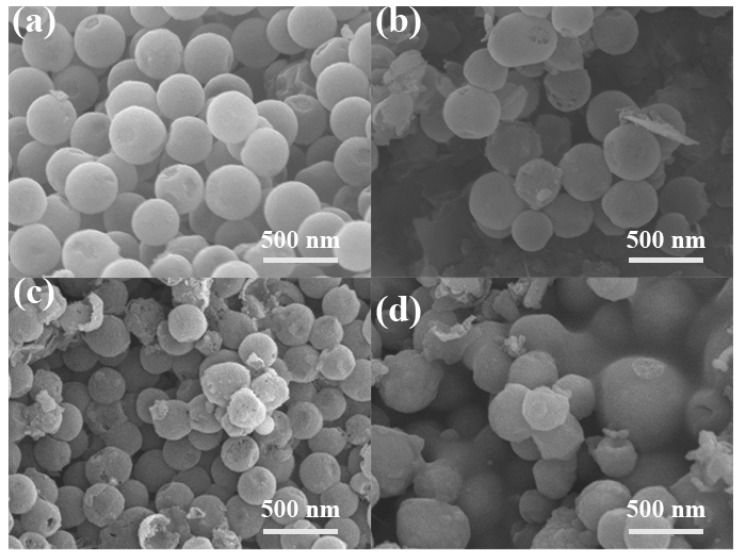
SEM images of (**a**) hollow N-doped carbon nanosphere (HNCS); (**b**) Fe@HNCS-800; (**c**) Fe@HNCS-900; and (**d**) Fe@HNCS-1000.

The SEM images of HNCS, Fe@HNCS-800, Fe@HNCS-900, and Fe@HNCS-1000 are displayed in Figure 2. It is clear that the HNCS exist as regular spheres, which have a uniform particle size of around 250 nm, while sparse pores existed on the nanospheres walls (Figure 2a). However, numerous broken debris can clearly be observed in Fe@HNCS-800 (Figure 2b). These broken nanospheres are believed to be a result of the aggressive reaction between the trapped FeCl_3_ and the carbon spheres, which could take place during the pyrolysis process [51]. More broken nanospheres could be observed at higher pyrolysis temperatures, suggesting more reactions between the carbon walls and the iron species (Figure 2b–d). The hollow structure is clearly revealed in the partially broken nanospheres, as shown in Figure 2c.

**Figure 3:**
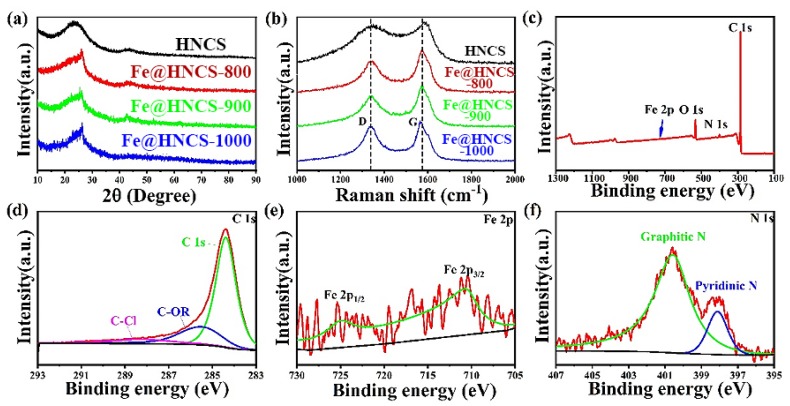
(**a**) X-ray diffraction (XRD) patterns and (**b**) Raman spectra of all samples; (**c**) X-ray photoelectrons spectroscopy (XPS) survey spectrum of Fe@HNCS-800; high-resolution spectra of (**d**) C 1s peak, (**e**) Fe 2p peak, and (**f**) N 1s peak.

In order to study the crystallinity and phase purity of all products, X-ray diffraction (XRD) patterns were acquired, as shown in Figure 3a. A broaden peak occurred in the area ranging from 20° to 30° and can be observed in all patterns, indicating that all the samples are dominated by amorphous carbon [52,53]. Notably, the broad peak becomes narrower after the addition of Fe^3+^, suggesting some growth of crystallites in the lateral direction. The slight increase in the carbon crystallinity can be attributed to the catalytic effect of the metallic iron derived from FeCl_3_, which promotes graphitization during the calcination [48]. No peaks were observed in the XRD patterns for any of the Fe species, such as iron oxide or iron carbide, which can be explained by the low loading of iron in the samples. Raman spectroscopy was used to characterize the microstructure further and is presented in Figure 3b. Two peaks centered at 1350 and 1590 cm^−1^ can be clearly observed in the spectra, which are known as D band and G band. In general, the G band is ascribed to the motion of an in-plane bond stretching of sp^2^ hybridized carbon atoms, whereas the D band corresponds to the breathing mode of the six-fold aromatic ring near the basal edge [52,54,55,56]. These results suggest that all products are hard carbons with a characteristic of graphite-like layered structure but without stacking between the layers. The results of both XRD and Raman analysis indicate that there are no significant changes in the microstructure that occurred when the pyrolysis temperature increased from 800 °C to 1000 °C.

X-ray photoelectrons spectroscopy (XPS) was used to study the chemical state and composition of all Fe@HNCS-*T* samples. The XPS survey spectrum of Fe@HNCS-800 indicated the presence of Fe, O, N, and C in the sample (Figure 3c), and spectra of other products are similar (not showing here). The C 1s spectrum of Fe@HNCS-800 shows a broad peak between 283 eV and 287 eV (Figure 3d), which can be deconvoluted into three peaks at 284.5 eV, 285.5 eV, and 288.3 eV, corresponding to C-C, C-OR, and C-Cl, respectively [57]. The N 1s spectrum is shown in Figure 3f. The two deconvoluted peaks at 400.6 eV and 398.1 eV are attributed to graphitic and pyridinic N [58]. The Fe 2p spectrum showed two peaks at 711 eV and 725 eV, suggesting the existence of Fe^2+^ and Fe^3+^ (Figure 3e) [51]. It demonstrated that Fe ions only coordinated with N and no metallic Fe (706.5 eV) exists in Fe@HNCS-800 [59]. The atomic ratio of Fe in Fe@HNCS-800 is 0.14%. Previous studies suggested that atomic metals are hard to form in carbon without any heteroatoms [60]. The pyridinic N, which existed at the edges of the carbon basal planes, enabled coordination with Fe ions so that Fe ions could be firmly bonded in the carbon nanosphere [34]. Moreover, the intensities of Fe and pyridinic N peaks, namely their contents, decreased with increasing pyrolysis temperature (Figure S1, Supporting Information). Specifically, the atomic contents of Fe are 0.14% for Fe@HNCS-800, 0.1% for Fe@HNCS-900, and 0.08% for Fe@HNCS-1000; and the corresponding atomic contents of pyridinic N are 0.44%, 0.3%, and 0.2%, respectively.

**Figure 4:**
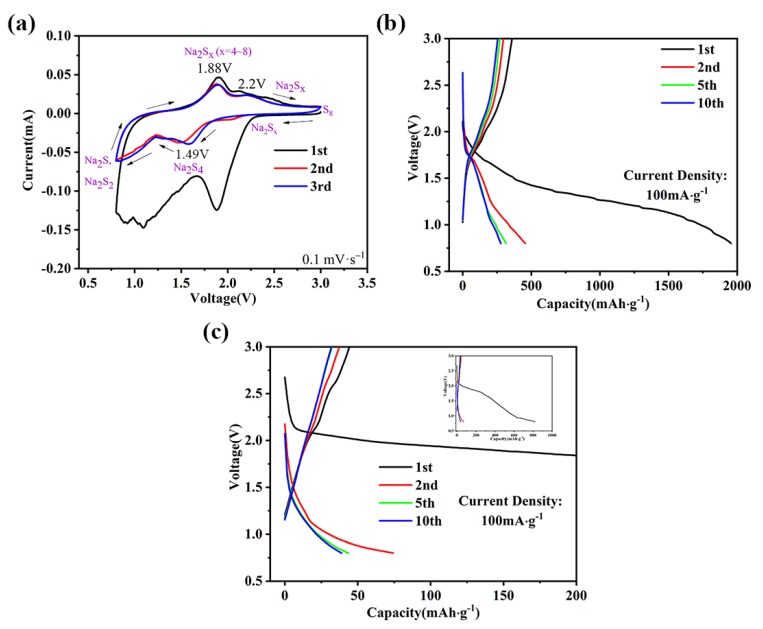
(**a**) Cyclic voltammograms (CV) curves of Fe@HNCS-800. GCD curves of (**b**) Fe@HNCS-800 and (**c**) HNCS. The inset of (**c**) is the full range GCD curve of HNCS.

To investigate the electrochemical performance of the Fe@HNCS-*T* composites, S was loaded into Fe@HNCS with a weight ratio of 6:4 and used as the cathode for the RT Na-S battery. The electrolyte used was bis(trifluoromethane)-sulfonimide sodium (NaTFSI) in propylene carbonate (PC) and fluoroethylene carbonate (FEC) as cosolvents. Selection of the electrolyte was based on its reported excellent electrochemical performance, particularly its ability to suppress the shuttle effect [61]. The cyclic voltammograms (CV) of the HCNS and Fe@HNCS-800 electrodes were acquired at a scan rate of 0.1 mV·s^−1^ (Figure S2a and Figure 4a). The CV for HNCS shows two cathodic peaks at 1.28 V and 1.8 V corresponding to the conversion of S to high-order polysulfides and the reduction of the high-order polysulfides to low-order sulfides [27,62]. Similar peaks can be observed for the Fe@HNCS-800 electrode, with the positions of the peaks slightly shifted toward more positive potentials (1.49 V and 2 V). Shifts of the peak potential were also observed for anodic peaks which originated from the oxidation of sulfides to S_8_. The two anodic peaks which were located at 2.09 V and 2.52 V in the CV curve of the HNCS electrode shifted negatively to 1.88 V and 2.2 V for Fe@HNCS-800. Previous studies have demonstrated that these shifts of peak potentials [33,34,35], including both the positive shift for cathodic peaks and negative shift for anodic peaks, and the increase in the current in the Fe@HNCS-800 electrode were associated with the catalytic activity of Fe ions during the conversion reaction of S. Redox peaks for S were still clearly seen at the third cycle of the Fe@HNCS-800 electrode. In contrast, the redox peaks of S almost vanished after the third cycle of the HNCS electrode (Figure S2), suggesting that the iron-free electrode could not restrict the diffusion of polysulfides during the charge and discharge process. These results manifest that the “shuttle effect” was effectively suppressed in Fe@HNCS-800, which can be explained by the fact that the Fe ions accelerate the transition of long-chain polysulfide to short-chain sulfide and also possess strong chemical adsorption ability with polysulfides [34,35].

The profiles of galvanostatic charge–discharge (GCD) coincide with the CV curves. Figure 4b,c shows GCD profiles of the 1st, 2nd, 5th and 10th cycles of the Fe@HNCS-800 and HNCS electrodes at 0.1 A g^−1^. The HNCS electrode only showed a reversible capacity of 45 mAh·g^−1^ after the first cycle, and no clear plateau was observed at the fifth cycle, suggesting all active materials are consumed during the charge and discharge process. On the contrary, two plateaus that span from 1.85 V to 1.25 V and 1.25 V to 0.8 V were clearly observed during the discharge process of the Fe@HNCS-800 electrodes, corresponding to the two cathodic peaks in the CV curves, respectively. In the first discharge and charge process, high discharge capacity (~2000 mAh·g^−1^) and charge capacity (359 mAh·g^−1^) for Fe@HNCS-800 with an initial coulomb efficient (ICE) of 17.95% were observed. Such a capacity loss originates from the electrolyte decomposition, the side reaction between the carbonate-based solvents and soluble polysulfides, and the formation of the solid electrolyte interphase (SEI) films.

The voltage profiles of the HNCS and Fe@HNCS-800 electrodes at different densities are also presented in Figure S3. The polarization potentials of Fe@HNCS-800 increased from 170 mV at 0.1 A·g^−1^ to 510 mV at 1 A·g^−1^. A higher polarization potential at higher currents suggests lower reversibility for the redox reaction. However, this value is still much lower than that of HNCS (1000 mV at 0.1 A·g^−1^). These results suggest that incorporating Fe into N-doped carbon nanospheres, even at an extremely low content, is an effective approach to enhance the performance of the RT Na-S battery.

**Figure 5:**
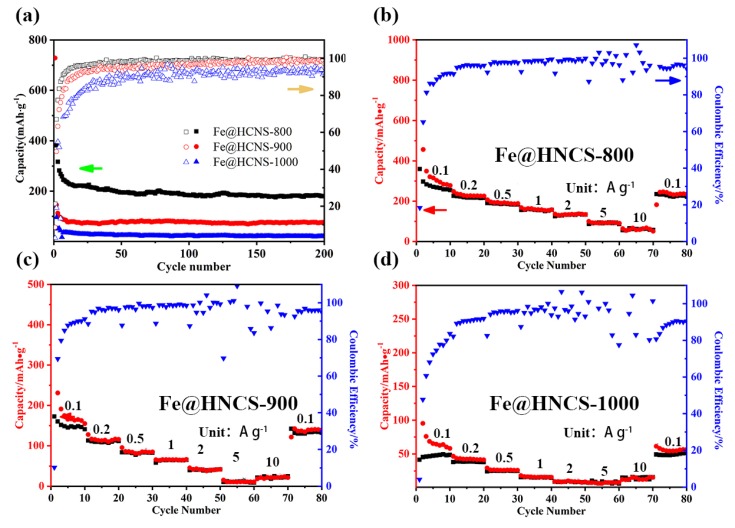
(**a**) Cyclic performance of Fe@HNCS-800, Fe@HNCS-900, and Fe@HNCS-1000; (**b**) Rate performance of Fe@HNCS-800; (**c**) Rate performance of Fe@HNCS-900; and (**d**) Rate performance of Fe@HNCS-1000.

Good cyclic stability is crucial for practical applications of host material for the RT Na-S battery. The cycling performance of the HNCS, Fe@HNCS-800, Fe@HNCS-900, and Fe@HNCS-1000 electrodes were tested at a current density of 1 A·g^−1^ (Figure S4a and Figure 5a). All the Fe-containing samples (carbonized at different temperatures) became stable after 10 cycles. It can be seen that the HCNS electrode lost almost all the capacity after 200 cycles (Figure S4a). On the contrary, the Fe@HNCS-800 electrode maintains a charge capacity of 180 mAh·g^−1^ after 200 cycles with a coulombic efficiency close to 100% during the cycling process, further confirming that the electrocatalytic effect of Fe ions during the repetitive redox reaction of S resulted in better management of polysulfides. Furthermore, the strong interactions between the Fe ions and polysulfides suppressed the dissolution of polysulfides into the organic electrolyte, which has been demonstrated in previous reports [34,35]. These effects led to a significant enhancement in the reversible capacity of the Fe@HNCS-800 electrode. It is also noted that the reversible capacity decreases with increasing pyrolysis temperature (~100 mAh·g^−1^ for Fe@HNCS-900 and ~30 mAh·g^−1^ for Fe@HNCS-1000). This can be explained by the decreasing contents of Fe ions and pyridinic N with the increasing pyrolysis temperature, and thus a reduction of the catalytic activity and weaker chemical absorption ability of polysulfides.

The rate performance was also studied by using a repetitive GCD process at different current densities ranging from 0.1 A·g^−1^ to 10 A·g^−1^, charging and discharging for 10 cycles at each current, as shown in Figure S4b and Figure 5b–d. The charge capacities of HNCS are 38.9, 23.4, 6.6, 2.6, and 3.6 mAh·g^−1^ at current densities of 0.1, 0.2, 0.5, 1, and 2 A·g^−1^. In addition, the battery assembled using the HNCS electrode could not charge and discharge at a current density higher than 5 A·g^−1^ due to the sluggish reactivity of S with Na. In contrast, the charge capacities of the Fe@HNCS-800 electrode at the 10th cycle at each charge and discharge step were 254.9, 216.3, 183.4, 156.6, 133.7, 86.5, and 56.9 mAh·g^−1^ at current densities of 0.1, 0.2, 0.5, 1, 2, 5, and 10 A·g^−1^, respectively, i.e., the capacity retained 22.3% of its initial value when the current density was increased by 100 times. Strikingly, when the current density was finally reverted to 0.1 A·g^−1^, a reversible capacity of 235.4 mAh·g^−1^ was recovered. These results confirmed the excellent rate performance of Fe@HNCS-800. Moreover, the capacities of Fe@HNCS-800 are higher than those of Fe@HNCS-900 and Fe@HNCS-1000 at all tested currents. The best performance of Fe@HNCS-800 among these samples can be attributed to the highest content of pyridinic N and consequently the content of Fe, which in turn exhibits the highest catalytic activity towards the conversion of polysulfides and stronger interaction with soluble polysulfides.

## 3. Experimental

### 3.1. Preparation of HNCS and Fe@HNCS

The synthesis processes of HNCS and Fe@HNCS are shown in Figure 1. First, 1 mL tetraethyl orthosilicate (TEOS, Macklin, Shanghai, China, 98%) and 8 mL dopamine hydrochloride (Sigma, St. Louis, MI, USA 98%) solution (50 mg·mL^−1^) were added sequentially and drop-by-drop along with continuous stirring to a mixed solution containing 80 mL deionized water, 24 mL ethanol (C_2_H_5_OH, Guangdong Guanghua Sci-Tech Co., Ltd, 99%), and 1 mL ammonium hydroxide (NH_3_·H_2_O, Guangdong Guanghua Sci-Tech Co., Ltd, 25~27%) at room temperature. Then, the suspension was centrifuged after stirring for 24 h, washed for three times using deionized water, and then three times using C_2_H_5_OH, and dried in the oven overnight to give rise to PDA@SiO_2_. Then, 0.4 g PDA@SiO_2_ was dispersed in an aqueous solution containing 0.8 g FeCl_3_ (Macklin, CP) under ultrasonication. The mixture was dried in the oven overnight, and then carbonized at different temperatures (800 °C, 900 °C, and 1000 °C) under argon (Ar) atmosphere, followed by washing with 10 wt.% HCl (Sinopharm Chemical Reagent Co., Ltd, 36~38%) solution for 12 h to remove large Fe particles. Then, the SiO_2_ template was removed from the products (Fe-PDA@SiO_2_) using a 2M KOH (Macklin, 95%) solution at 80 °C for 24 h. Finally, the as-synthesized samples were calcined again at 800 °C for 2 h under Ar atmosphere to achieve Fe@HNCS-*T*. For comparison, PDA@SiO_2_ was carbonized at 800 °C, washed with HCl solution, template removed with KOH, and further calcined under the same conditions to obtain HNCS.

### 3.2. Characterization

XRD patterns of the HNCS and Fe@HNCS-*T* were acquired using a PANalytical Empyrean X-ray diffractometer (Malvern Panalytical Ltd, Almelo, The Netherland) with Cu K_α_ radiation at 45 kV and 40 mA. The morphology of the HNCS and Fe@HNCS-*T* was observed by field emission scanning electron microscopy (JSM-7800F&TEAM Octane plus, JEOL, Tokyo, Japan). XPS data were collected using a Thermo Fisher K-Alpha+ instrument (Thermo Fisher Scientific Inc, Waltham, MA, USA). Renishaw inVia (Renishaw plc, London, UK) instrument was used to perform Raman spectroscopic study of the samples.

### 3.3. Electrochemical Measurement

The melt-diffusion method was used to combine the as-prepared samples with S (Sigma, S/sample = 6:4, the ratio by weight) at 155 °C using carbon disulfide as a solvent. To prepare the working electrodes, the active material, acetylene black (AB, MTI Corporation) and polymeric binder (polyvinylidene difluoride, PVDF, MTI Corporation), were mixed in a weight ratio of 8:1:1 using *N*-methyl-2-pyrrolidone (NMP, Aladdin) as a solvent to make a slurry. The obtained slurry was covered with an aluminum foil and dried under vacuum overnight. Then, the working electrodes were cut into a circular shape. The electrochemical properties were measured using CR2032 coin-type batteries, which were assembled using Na metal as the counter and reference electrodes in a glove box under Ar atmosphere (MBRAUN, UNILab2000, both moisture and oxygen level below 1 ppm). A glass fiber separator (Whatman GF/F) was used as the separator. A 2M NaTFSI electrolyte prepared by PC/FEC (DoDoChem) mixed in 1:1 volume ratio was used in the RT Na-S battery. The galvanostatic charge–discharge analysis was carried out in the range of 0.8 V to 3.0 V (vs. Na/Na^+^) on a battery test system (CT-2001A, LAND Electronic Co.Ltd, Wuhan, China). The capacity was calculated on the basis of S weight in the electrode throughout the full process. Cyclic voltammetry analysis was performed using an electrochemical workstation (CHI760D, Shanghai China) at a scan rate of 0.1 mV·s^−1^ from 0.8 to 3.0 V (vs. Na/Na^+^).

## 4. Conclusions

In summary, we developed a novel host material that contained Fe ions in hollow N-doped carbon nanospheres and enhanced the electrochemical performance of the RT Na-S battery. The Fe ions improve the chemical absorption of polysulfide and reaction kinetics, whereas the carbon framework acts as a continuous transport pathway. This multifunctional host material was therefore confirmed to be effective in the encapsulation of polysulfides and endowed improved kinetics of the polysulfide redox reactions. In addition, the effect of pyrolysis temperature on electrochemical properties was studied. The results show that the multifunctional host material obtained at 800 °C presented the highest reversible capacity (359 mAh·g^−1^ at 0.1 A·g^−1^), stable cycling performance (180 mAh·g^−1^ after 200 cycles with near 100% coulombic efficiency), and the best rate performance. The approach of using an electrocatalyst to suppress the shuttle effect and increase the chemical adsorption of polysulfides should pave the way for developing high-performance electrode materials to meet the future demands on Na-S batteries.

## Supplementary Materials

The following are available online, Figure S1: High resolution XPS spectrums of (a) Fe 2p, (b) N 1s for Fe@HNCS-900, (c) Fe 2p and (d) N 1s for Fe@HNCS-1000, Figure S2: CV curves of HCNS, Figure S3: GCD profiles of (a) HNCS, (b) Fe@HNCS-800 tested at different current density, Figure S4 (a) cycle performance and (b) rate performance of HCNS.
