# Supplementary material for: Electrocatalytic Assisted Performance Enhancement for the Na-S Battery in Nitrogen-Doped Carbon Nanospheres Loaded with Fe
Source: Molecules. 2020 Mar 30;25(7):1585. doi: 10.3390/molecules25071585 (PMC7180711; doi:10.3390/molecules25071585)
Supplement: Supplementary file 1 [file molecules-25-01585-s001.pdf]

Supporting information

Electrocatalytic assisted performance enhancement for Na-S battery in nitrogen-doped carbon nanospheres loaded with Fe

Jianhui Zhu [a,b], Amr Abdelkader [c], Denisa Demko [d], Libo Deng*[a], Peixin Zhang *[a,b], Tingshu He [b], Yanyi Wang [a,b] and Licong Huang [a]

[a] College of Chemistry and Environmental Engineering, Shenzhen University, Shenzhen 518060, China

[b] College of Materials Science and Engineering, Xi'an University of Architecture and Technology, Xi'an, Shannxi 710055, PR China

[c] Department of Design and Engineering, Faculty of Science & Technology, Bournemouth University, Poole, Dorset BH12 5BB, United Kingdom

[d] Department of Engineering, University of Cambridge, Cambridge CB3 0FS, United Kingdom

*Corresponding authors: Denglb@szu.edu.cn; pxzhang@szu.edu.cn

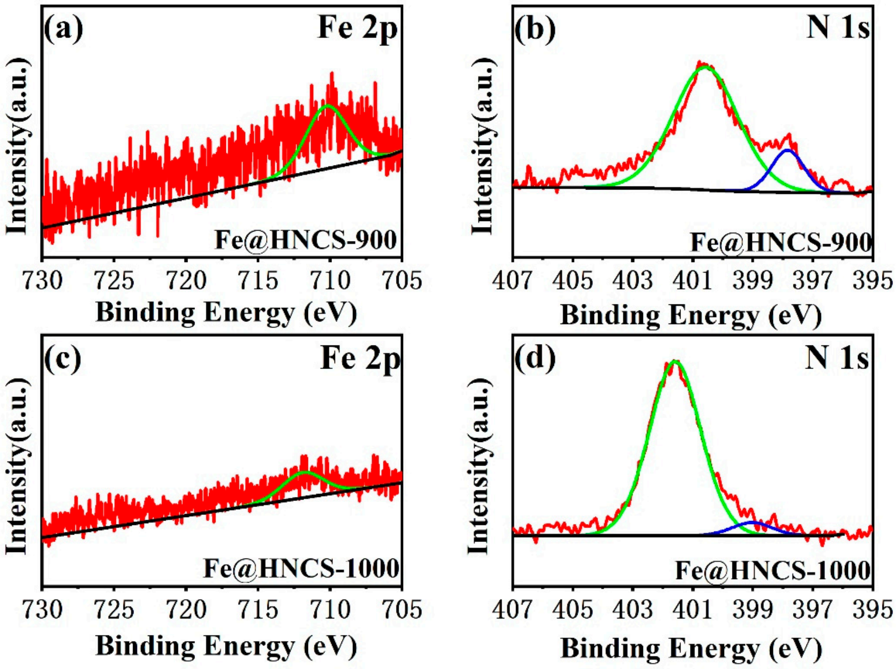

Fig. S1 High resolution XPS spectra of (a) Fe 2p, (b) N 1s for Fe@HNCS-900, (c) Fe 2p and (d) N 1s for Fe@HNCS-1000.

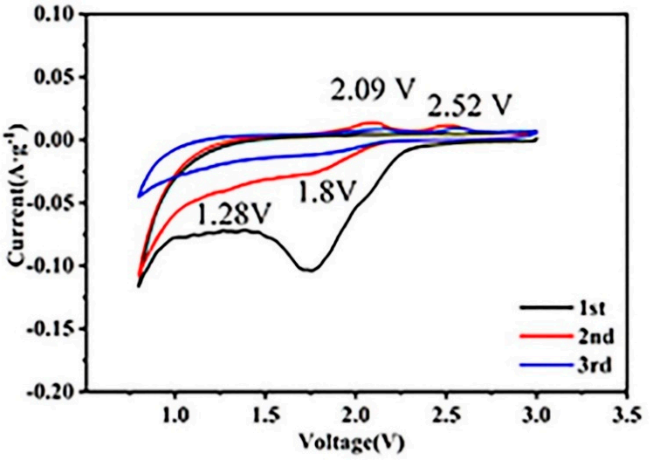

Fig. S2 CV curves of Fe@CNS-800

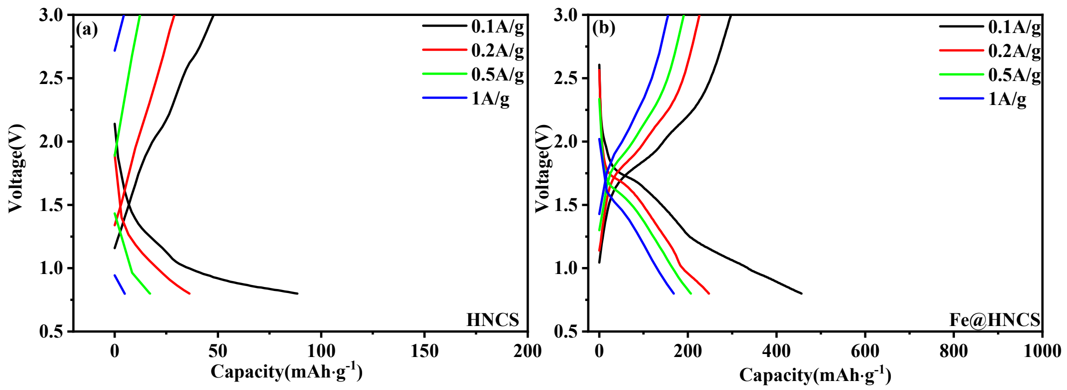

Fig. S3 GCD profiles of (a) HNCS, (b) Fe@HNCS-800 tested at different current densities

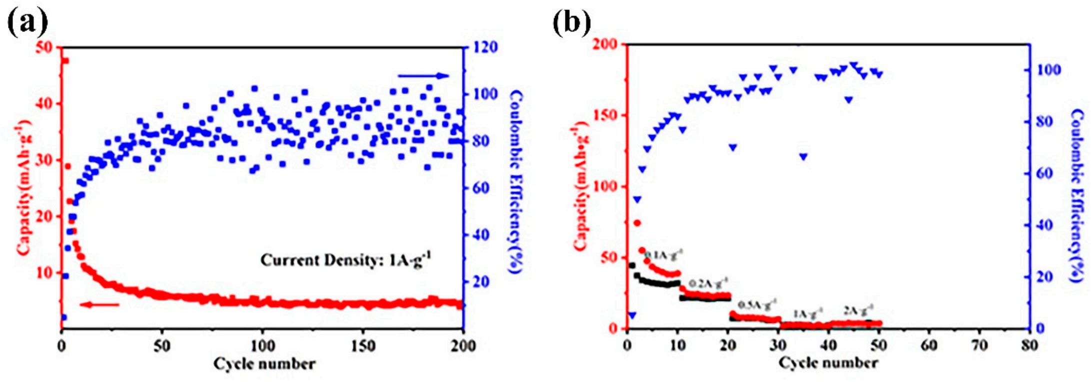

Fig. S4 (a) cyclic performance and (b) rate performance of HCNS.
